# Osaka Prognostic Score Predicts In-Hospital and One-Year Mortality Following Transcatheter Aortic Valve Implantation

**DOI:** 10.3390/medicina61122142

**Published:** 2025-11-30

**Authors:** Murat Akdoğan, Emine Cansu Yücel, Alperen Taş, Yusuf Bozkurt Şahin, Çağatay Tunca, Ahmet Kıvrak, Kürşat Akbuğa, Veysel Ozan Tanık, Ali Sezgin, Bülent Özlek

**Affiliations:** 1 Department of Cardiology, Ankara Etlik City Hospital, 06000 Ankara, Turkey; mrtakdgn66@gmail.com (M.A.); cnsyclcy@gmail.com (E.C.Y.); ybozkurtsahin@gmail.com (Y.B.Ş.); md.tunca@gmail.com (Ç.T.); akbuga_1453@hotmail.com (K.A.); drozantanik@gmail.com (V.O.T.); ali_sezgin_666@hotmail.com (A.S.); 2 Department of Cardiology, Kırşehir Training and Research Hospital, 40080 Kırşehir, Turkey; alperentas555@hotmail.com; 3 Department of Cardiology, School of Medicine, Hacettepe University, 06000 Ankara, Turkey; a.kivrak89@gmail.com; 4 Department of Cardiology, School of Medicine, Muğla Sıtkı Koçman University, 48999 Muğla, Turkey

**Keywords:** all-cause mortality, inflammation, malnutrition, Osaka prognostic score, transcatheter aortic valve implantation

## Abstract

*Background and Objectives*: Conventional risk models may not adequately capture key biological determinants of mortality following transcatheter aortic valve implantation (TAVI), such as inflammation, malnutrition, and immune dysfunction. The Osaka Prognostic Score (OPS), incorporating CRP, albumin, and lymphocyte count, may address this gap. We aimed to evaluate the prognostic value of OPS for predicting in-hospital and one-year all-cause mortality after TAVI. *Materials and Methods*: In this retrospective single-center cohort study, 244 patients who underwent transfemoral TAVI between December 2022 and January 2024 were analyzed. OPS was calculated at baseline (range: 0–3), and its prognostic value for in-hospital and one-year all-cause mortality was evaluated using multivariable Cox regression, ROC analysis, and restricted cubic spline (RCS) modeling. *Results*: In-hospital and one-year mortality rates were 11.5% and 21.7%, respectively. Higher OPS scores were significantly associated with mortality in both periods. OPS independently predicted in-hospital mortality (HR: 2.018; 95% CI: 1.632–2.521; *p* = 0.017) and one-year mortality (HR: 2.125; 95% CI: 1.300–3.473; *p* = 0.003). ROC analysis yielded AUC values of 0.776 and 0.859 for in-hospital and one-year mortality, respectively. Kaplan–Meier curves revealed significantly reduced survival in patients with higher OPS (log-rank *p* < 0.001), and RCS analysis demonstrated a significant nonlinear association between increasing OPS values and mortality risk (*p* < 0.001). *Conclusions*: OPS is a simple, cost-effective, and biologically relevant prognostic index that independently predicts both in-hospital and one-year mortality following TAVI. By integrating markers of inflammation, nutrition, and immune competence, OPS may offer additional value in risk stratification and support clinical decision-making in this high-risk population.

## 1. Introduction

Transcatheter aortic valve implantation (TAVI) has emerged as a cornerstone therapy for patients with severe aortic stenosis (AS) who are considered at high or prohibitive risk for surgical aortic valve replacement (SAVR) [1]. Over the past decade, refinements in procedural techniques and more judicious patient selection have significantly enhanced the safety and efficacy of TAVI [2]. Nevertheless, short- and long-term mortality rates remain unacceptably high in certain subgroups, particularly among those with frailty, multiple comorbidities, or elevated systemic inflammatory burden [3]. Accordingly, there is a growing need for reliable, easily accessible prognostic markers to guide clinical decision-making and optimize outcomes in this complex population. Contemporary European guidelines emphasize the use of established surgical risk scores, such as the Society of Thoracic Surgeons Predicted Risk of Mortality (STS-PROM) and EuroSCORE-II, to inform the decision between TAVI and SAVR, alongside patient age and operative risk profiles [3]. However, these models were originally developed for patients undergoing conventional cardiac surgery and may not fully encapsulate the unique prognostic determinants relevant to TAVI candidates. Moreover, their reliance on complex variables and lack of integration of inflammatory or nutritional indices may limit their applicability in real-world settings. There is, thus, a compelling rationale for exploring simpler, more pragmatic tools that incorporate biological parameters reflective of systemic health.

Systemic inflammation, malnutrition, and immunosuppression are increasingly recognized as central drivers of adverse outcomes in cardiovascular disease, including among patients undergoing TAVI [4,5,6]. In this context, emerging composite indices, such as the Osaka Prognostic Score (OPS), have attracted attention. Originally developed to predict survival in oncology populations, the OPS integrates three readily available and cost-effective biomarkers: C-reactive protein (CRP), albumin, and total lymphocyte count (TLC) [7]. These variables collectively reflect inflammatory status, nutritional reserves, and immune competence—all of which are known to influence both perioperative resilience and long-term prognosis in cardiovascular patients [8,9,10]. Elevated CRP levels, a hallmark of systemic inflammation, have been independently associated with increased mortality following TAVI [11]. Likewise, hypoalbuminemia, reflecting both malnutrition and chronic inflammation, has been linked with impaired procedural outcomes and survival [12]. Lymphopenia, as a surrogate marker of immune dysfunction, also portends a worse prognosis in multiple clinical contexts [10]. Given this pathophysiological interplay, integrating these parameters into a unified prognostic tool, such as the OPS, may provide a more holistic and clinically relevant risk assessment.

Therefore, the present study aims to investigate the prognostic value of the OPS in predicting both in-hospital and one-year all-cause mortality among patients undergoing TAVI. We further seek to evaluate the feasibility of incorporating this score into routine clinical practice as a simple, inexpensive, and biologically grounded tool for risk stratification in this high-risk population.

## 2. Materials and Methods

### 2.1. Study Design and Population

This retrospective, single-center cohort study included consecutive patients with severe symptomatic AS who underwent TAVI between December 2022 and January 2024 at a tertiary cardiovascular center. Baseline demographic, clinical, laboratory, and procedural data were retrieved from the institution’s electronic medical record system. The diagnosis of comorbidities—including hypertension, diabetes mellitus (DM), and chronic obstructive pulmonary disease (COPD)—was based on standard clinical definitions supported by medical documentation. Hypertension was defined as a systolic blood pressure ≥ 140 mmHg, a diastolic pressure ≥ 90 mmHg, or ongoing antihypertensive treatment. DM was diagnosed based on a fasting plasma glucose level > 126 mg/dL, an HbA1c ≥ 6.5%, or the use of glucose-lowering agents. COPD was confirmed via International Classification of Diseases (ICD) codes and prior pulmonology evaluations. Contrast-induced nephropathy (CIN) was defined as an absolute increase in serum creatinine of ≥0.5 mg/dL or a relative increase of ≥25% from baseline within 48 h after contrast administration in the absence of alternative causes [13].

Exclusion criteria included advanced hepatic dysfunction (Child–Pugh class B or C), active systemic infections, sepsis, autoimmune or connective tissue diseases, active malignancy under treatment, and patients undergoing emergent or bailout TAVI procedures. Laboratory tests—including complete blood count, serum biochemistry, and inflammatory markers—were obtained within 24 h of hospital admission. Of the 254 patients initially screened, 244 met all eligibility criteria and were included in the final analysis.

The primary outcome of the study was all-cause mortality occurring during the initial hospitalization and at 1-year follow-up after the TAVI procedure.

The study protocol was approved by the local institutional ethics committee (Ankara Etlik City Hospital, approval date and number: 22 May 2024–AEŞH-BADEK-2024-472) and conducted in accordance with the ethical principles outlined in the Declaration of Helsinki. Given the retrospective design, informed consent was waived.

### 2.2. TAVI Procedure and Periprocedural Imaging

The diagnosis of severe AS was established through transthoracic echocardiography based on an aortic valve area < 1.0 cm^2^ or a mean transvalvular gradient ≥ 40 mmHg in the presence of compatible clinical symptoms [3]. All patients were evaluated preoperatively by a multidisciplinary heart team comprising interventional cardiologists, cardiac surgeons, and imaging specialists to determine procedural eligibility. All TAVI procedures were performed by experienced interventional cardiologists using a transfemoral approach in a hybrid operating room or a catheterization laboratory. Conscious or deep sedation was administered based on clinical judgment. Procedural guidance was provided by real-time transthoracic echocardiography and electrocardiography, supplemented with pre-procedural computed tomography (CT) for anatomical assessment and valve sizing. Unfractionated heparin was administered to achieve target anticoagulation levels during the procedure. Rapid ventricular pacing was utilized during valve deployment to minimize motion. Vascular closure was achieved using dedicated closure devices, such as Perclose ProGlide (Abbott Vascular, Santa Clara, CA, USA) or AngioSeal (St. Jude Medical, St. Paul, MN, USA), at the operator’s discretion. The choice of prosthetic valve was individualized based on anatomical and procedural considerations, including balloon-expandable valves (e.g., Edwards SAPIEN S3 and XT, Myval) and self-expanding valves (e.g., Medtronic Evolut R, Portico, and LOTUS). Valve sizing adhered to manufacturer-specific protocols. Post-procedural monitoring was conducted in an intensive care or step-down unit, and complications were recorded according to the standardized definitions of the Valve Academic Research Consortium-3 [14]. Surviving patients were discharged on optimal medical therapy and scheduled for outpatient follow-up at 1, 6, and 12 months.

### 2.3. Definition of Prognostic Indices

The OPS was calculated using three routinely available laboratory parameters: serum CRP, serum albumin, and TLC. One point was assigned for each of the following: CRP > 10.0 mg/L, albumin < 3.5 g/dL, and TLC < 1600/μL, yielding a total score ranging from 0 to 3 [15]. Patients were stratified into four OPS categories: 0, 1, 2, and 3 points. The Systemic Immune-Inflammation Index (SII) was calculated using the formula: platelet × neutrophil/lymphocyte [16].

### 2.4. Statistical Analysis

The distribution of continuous variables was assessed using the Kolmogorov–Smirnov test. Normally distributed variables were presented as the mean ± standard deviation, while non-normally distributed variables were expressed as median with interquartile ranges. Categorical variables were summarized as frequencies and percentages. Comparisons between groups were performed using the Student’s *t*-test or the Mann–Whitney U test for continuous variables and the chi-square or Fisher’s exact test for categorical variables, as appropriate. Univariable Cox proportional hazards regression analysis was conducted to identify factors associated with in-hospital and one-year mortality. Variables with *p*-values < 0.05 in univariable analysis were entered into multivariable Cox regression models using a forward stepwise selection method to identify independent predictors. Hazard ratios (HRs) with corresponding 95% confidence intervals (CIs) were reported. Given the limited number of 28 in-hospital events, multivariable modeling was restricted to the most clinically relevant and complementary pre- and peri-procedural predictors—age, vascular complications, and OPS—to construct a parsimonious, phenomenon-representative model while mitigating overfitting and collinearity. Although age and COPD showed a univariable association with one-year mortality, these variables were excluded from the multivariable Cox regression model because they are integral components of both the STS and EuroSCORE-II risk scores. Including them together with these composite indices could introduce multicollinearity and obscure the independent prognostic value of the scores. To verify this, multicollinearity diagnostics were performed using the variance inflation factor (VIF), which demonstrated high collinearity between age, COPD, and the composite risk scores (VIF > 5). Therefore, to maintain model stability and interpretability, age and COPD were omitted from the final model. The OPS served as the main variable of interest in both models. The discriminatory performance of OPS and other inflammatory indices was evaluated using receiver operating characteristic (ROC) curve analysis. The area under the curve (AUC), optimal cut-off values (determined by Youden’s index), sensitivity, and specificity were calculated for each predictor. AUC comparisons between correlated ROC curves were performed using the DeLong test. Survival analysis was performed using Kaplan–Meier curves stratified by OPS categories (0–3), and differences between groups were compared using the log-rank test. Additionally, to evaluate the potential nonlinearity in the association between OPS and mortality risk, restricted cubic spline (RCS) models were constructed. For in-hospital mortality, a logistic regression-based RCS model was applied, while a Cox regression-based spline model was used for one-year mortality. To assess the calibration performance of the OPS-based prediction models for in-hospital and one-year mortality, Hosmer–Lemeshow goodness-of-fit tests were performed. Calibration plots were generated by grouping patients into deciles based on predicted probabilities, and observed versus expected mortality rates were compared within each decile. A non-significant *p*-value from the Hosmer–Lemeshow test was interpreted as good agreement between predicted and observed outcomes. All statistical analyses were performed using SPSS version 26.0 (IBM Corp., Armonk, NY, USA) and Python (version 3.11) with relevant statistical libraries, including lifelines, stats models, and patsy. A two-sided *p*-value < 0.05 was considered statistically significant.

## 3. Results

### 3.1. Baseline Clinical Characteristics and In-Hospital Outcomes

A total of 244 patients who underwent TAVI were included in the final analysis. The median length of hospital stay was 10 days (range: 2–45 days). Based on the OPS, 86 patients (35.2%) had a score of 0, 99 patients (40.6%) had a score of 1, 40 patients (16.4%) had a score of 2, and 19 patients (7.8%) had a score of 3. Of these, 28 patients (11.5%) died during hospitalization. Compared with survivors, non-survivors were significantly older (85.6 ± 5.4 vs. 79.2 ± 8.2 years, *p* < 0.001). They had higher rates of COPD, vascular complications, and CIN. Laboratory results showed significantly lower hemoglobin, TLC, and albumin levels in non-survivors. In contrast, CRP levels were significantly higher. The OPS was markedly elevated in non-survivors (1.92 ± 0.99 vs. 0.80 ± 0.79, *p* < 0.001), and SII levels did not differ significantly between groups. Other demographic and clinical characteristics—including gender distribution, hypertension, DM, atrial fibrillation (AF), prior percutaneous coronary intervention (PCI) or coronary artery bypass graft (CABG), type of prosthetic valve, valve size, and echocardiographic parameters (left ventricular ejection fraction, aortic valve gradient, systolic pulmonary artery pressure)—were similar between groups (*p* > 0.05 for all). Medication use, including beta-blockers, angiotensin-converting enzyme inhibitors (ACEi) or angiotensin II receptor blockers (ARBs), statins, and antiplatelet agents, also did not differ significantly. Likewise, STS and EuroSCORE-II risk scores showed no statistically significant difference between survivors and non-survivors (Table 1).

### 3.2. Predictors of In-Hospital Mortality

**Figure 1:**
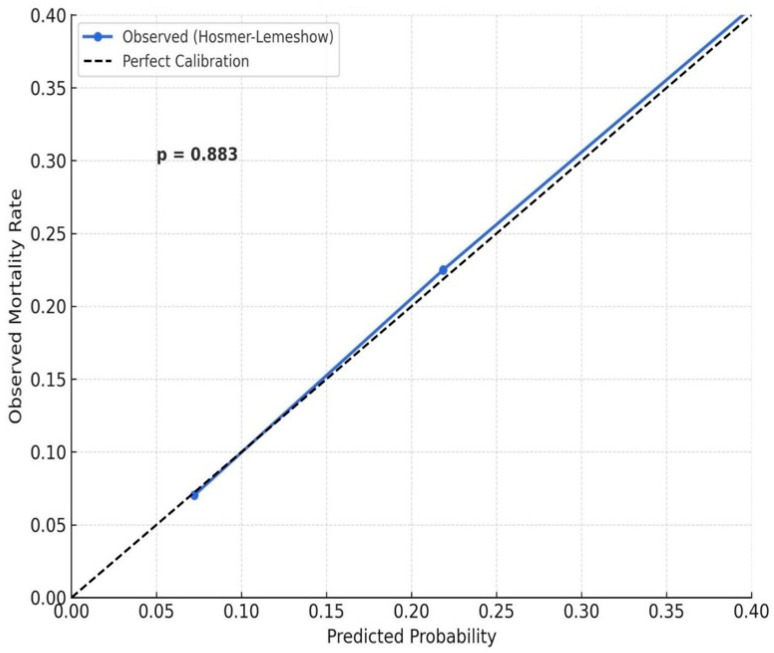
Hosmer–Lemeshow calibration plot for the OPS-based regression model predicting in-hospital mortality. The diagonal dashed line represents perfect calibration (i.e., predicted probabilities = observed outcomes). The blue line indicates the observed event rates across deciles of predicted risk. A non-significant *p*-value (*p* = 0.883) supports excellent model calibration.

**Figure 2:**
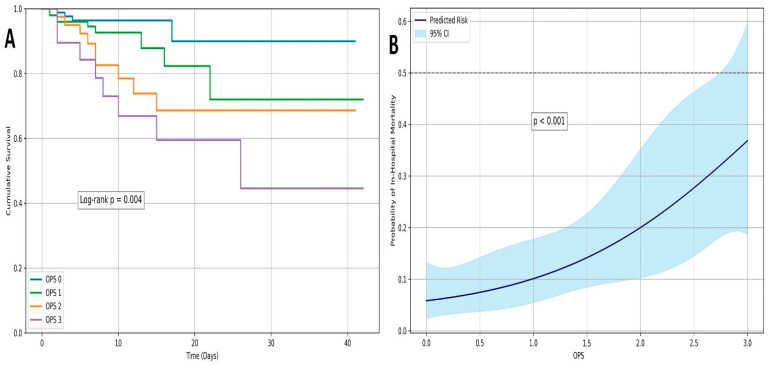
(**A**) Kaplan–Meier survival curves for in-hospital mortality stratified by Osaka Prognostic Score (OPS) groups. Patients with higher OPS values exhibited significantly lower short-term survival rates. The log-rank test demonstrated a statistically significant difference among the groups (log-rank *p* = 0.004); (**B**) Restricted cubic spline curve illustrating the nonlinear association between the OPS and in-hospital mortality. The spline model, derived from a generalized linear model with a binomial distribution, demonstrates a gradual increase in predicted mortality probability as OPS increases. The shaded area represents the 95% confidence interval. The association was statistically significant (*p* < 0.001).

In the univariable Cox regression analysis, advanced age, COPD, vascular complications, CIN, higher OPS, and lower hemoglobin were significantly associated with in-hospital mortality. In the multivariable model, OPS (HR: 2.018, 95% CI: 1.632–2.521, *p* = 0.017), age (HR: 1.821, 95% CI: 1.346–2.292, *p* = 0.020), and vascular complications (HR: 1.461, 95% CI: 1.208–1.657, *p* = 0.025) remained independent predictors (Table 2). ROC analysis demonstrated that the OPS had high discriminative power for in-hospital mortality with an AUC of 0.776 (*p* < 0.001), using a cut-off value of 2 (sensitivity: 64%, specificity: 82%). The Hosmer–Lemeshow goodness-of-fit test demonstrated excellent calibration for the OPS-based model (*p* = 0.883), complementing its high discriminative ability (Figure 1). Kaplan–Meier survival curves showed that patients with higher OPS values had significantly lower in-hospital survival rates (log-rank *p* = 0.004). In particular, the 40-day survival in the OPS 3 group was below 50%, whereas patients with OPS 0 demonstrated >90% survival (Figure 2A). Additionally, an RCS model demonstrated a progressive, nonlinear increase in in-hospital mortality probability with rising OPS scores (*p* < 0.001), further supporting its independent prognostic value (Figure 2B).

### 3.3. One-Year Outcomes and Baseline Characteristics

At one-year follow-up, 53 patients (21.7%) had died. Compared to survivors, non-survivors were significantly older (83.5 ± 6.8 vs. 79.0 ± 8.2 years, *p* < 0.001) and had a higher prevalence of COPD, vascular complications, and CIN. Non-survivors also had significantly higher perioperative risk scores, including STS (median 13.4 vs. 9.7, *p* < 0.001) and EuroSCORE-II (median 6.7 vs. 4.3, *p* < 0.001). The mean OPS was markedly elevated in the non-survivor group compared to survivors (1.84 ± 0.84 vs. 0.86 ± 0.75, *p* < 0.001). SII was also significantly higher in the one-year non-survivor group compared with survivors (*p* = 0.008). In addition, non-survivors had higher CRP levels, lower TLC, and lower albumin levels. No significant differences were observed between groups regarding sex, hypertension, DM, AF, valve type or size, or echocardiographic findings. Similarly, baseline medical therapy, including statins, ACEi/ARB, beta-blockers, and antiplatelet agents, was comparable (Table 3).

### 3.4. Predictors of One-Year Mortality

**Figure 3:**
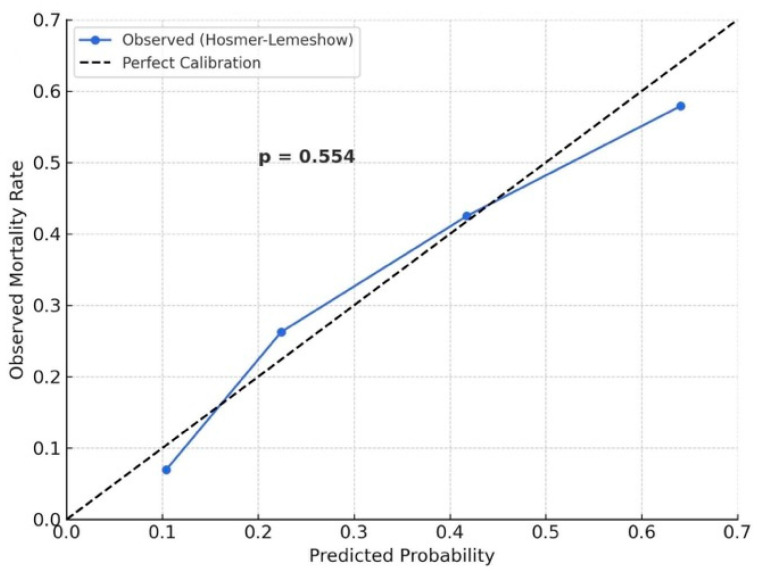
Hosmer–Lemeshow calibration plot for the OPS-based model predicting one-year all-cause mortality. Predicted mortality probabilities were divided into deciles, and observed versus expected mortality rates were plotted. The dashed line indicates perfect calibration. The close alignment between the observed and predicted rates (*p* = 0.554) indicates good model calibration.

**Figure 4:**
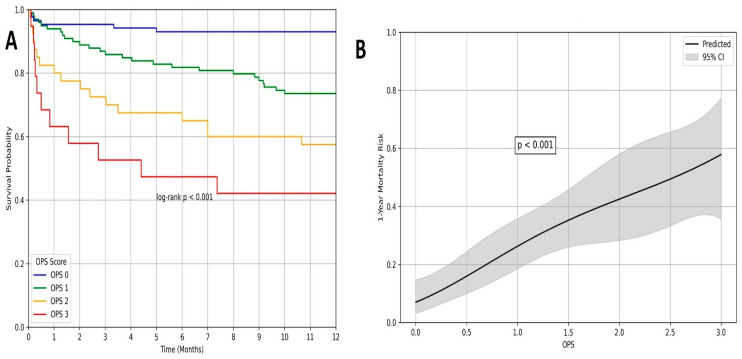
(**A**) Kaplan–Meier survival curves for one-year mortality stratified by Osaka Prognostic Score (OPS). Patients with higher OPS values had significantly lower one-year survival rates (log-rank *p* < 0.001). The OPS 3 group exhibited the poorest prognosis, with survival falling below 50%, while the OPS 0 patients had survival rates exceeding 90%. (**B**) Restricted cubic spline analysis illustrating the nonlinear association between the OPS and 1-year mortality risk in patients undergoing TAVI. The solid black line represents the predicted probability of death, and the shaded area indicates the 95% confidence interval. A significant incremental relationship was observed across increasing OPS values (*p* < 0.001).

In the univariable Cox regression analysis, several variables were significantly associated with one-year mortality, including advanced age, COPD, vascular complications, CIN, elevated STS score, EuroSCORE-II, and OPS. However, in the multivariable model, independent predictors of one-year mortality included OPS (HR: 2.125, 95% CI: 1.300–3.473, *p =* 0.003), vascular complication (HR: 1.202, 95% CI: 1.080–1.512, *p* = 0.001), and EuroSCORE-II (HR: 1.104, 95% CI: 1.006–1.211, *p* = 0.036) (Table 4). At one-year, OPS remained the prognostic indicator (AUC = 0.859, *p* < 0.001), using a cut-off of 2 (sensitivity: 44%, specificity: 87%). The calibration performance of the OPS-based predictive model for one-year mortality was further assessed using the Hosmer–Lemeshow goodness-of-fit test. The calibration plot revealed excellent agreement between predicted and observed mortality probabilities, with no significant deviation detected (*p* = 0.554), supporting the model’s reliability (Figure 3). Kaplan–Meier analysis revealed progressively lower survival across increasing OPS categories (log-rank *p* < 0.001). One-year survival in the OPS 3 group was <50%, whereas patients with OPS 0 had survival rates > 90% (Figure 4A). Furthermore, RCS regression analysis confirmed a significant nonlinear relationship between OPS and one-year mortality risk (*p* < 0.001), demonstrating a gradual increase in mortality probability with higher OPS values (Figure 4B).

### 3.5. Discriminatory Performance of OPS vs. Individual Biomarkers

**Figure 5:**
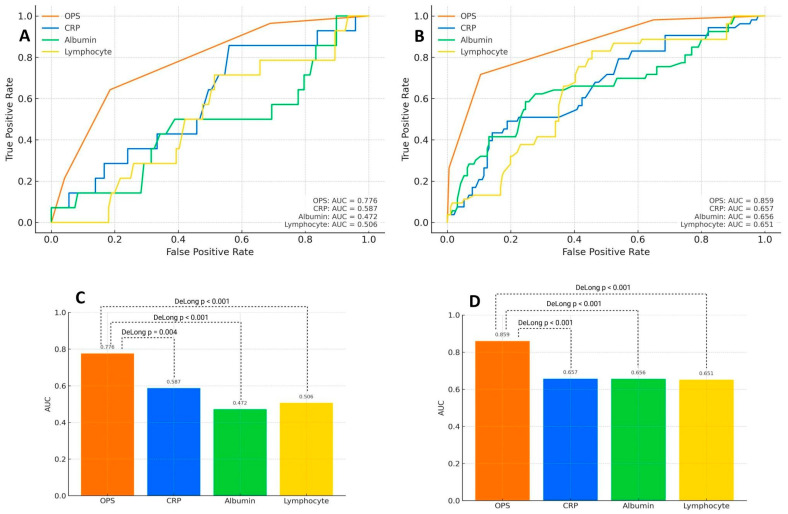
Prognostic performance of the Osaka Prognostic Score (OPS) and individual biomarkers for mortality after TAVI. (**A**) ROC curves for in-hospital mortality comparing OPS, C-reactive protein (CRP), serum albumin, and lymphocyte count. (**B**) ROC curves for one-year mortality comparing the same variables as in (**A**), using the identical color scheme. (**C**) The area under the curve (AUC) bar-chart for in-hospital mortality, illustrating the discriminative ability of OPS and individual biomarkers. (**D**) AUC bar-chart for one-year mortality, consistent with (**C**). AUC values for each model are displayed in the lower-right corner of (**A**,**B**). Statistical significance of correlated ROC comparisons was assessed using the DeLong test across (**C**,**D**).

ROC analysis showed moderate discrimination of the OPS composite score for in-hospital mortality, outperforming CRP alone, serum albumin, and TLC (Figure 5A). For one-year mortality, OPS demonstrated strong discrimination, exceeding CRP, albumin, and TLC alone (Figure 5B). DeLong test comparisons of correlated ROC curves demonstrated that OPS showed a statistically significant advantage over CRP alone for in-hospital mortality (Z = 2.84, *p* = 0.004; Figure 5C) and showed a strong trend toward improved discrimination for one-year mortality (Z = 4.41, *p* < 0.001; Figure 5D). OPS also demonstrated significantly superior discrimination compared with serum albumin alone (in-hospital: Z = 4.20, *p* < 0.001, Figure 5C; one-year: Z = 4.04, *p* < 0.001, Figure 5D) and compared with TLC alone (in-hospital: Z = 4.22, *p* < 0.001, Figure 5C; one-year: Z = 4.99, *p* < 0.001, Figure 5D). Collectively, these analyses indicate that OPS contributes incremental prognostic value beyond its individual components, supporting the rationale and added complexity of the composite score calculation.

**Figure 6:**
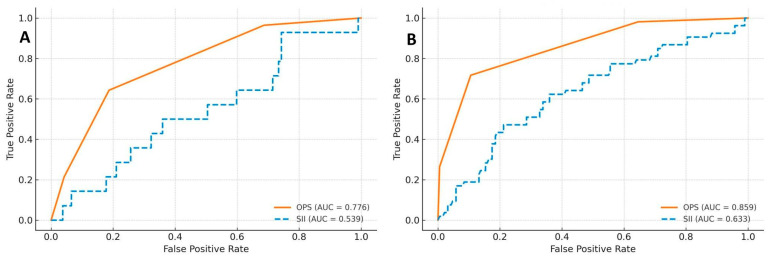

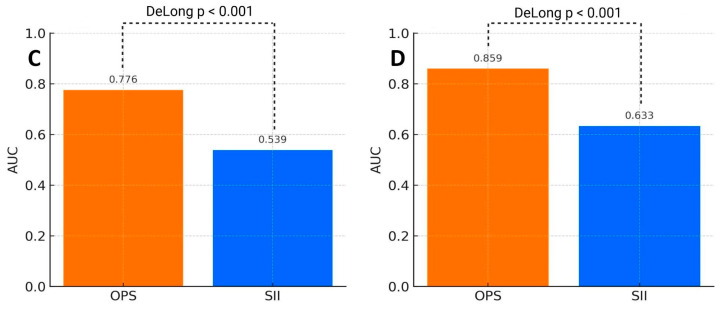
Composite figure comparing the prognostic performance of the Osaka Prognostic Score (OPS) composite index and the Systemic Immune-Inflammation Index (SII) for mortality prediction after TAVI. The figure contains paired ROC curves and corresponding AUC bar comparisons with DeLong significance testing. (**A**) In-hospital mortality ROC: OPS shows modest but superior discrimination over SII (AUC = 0.776 vs. 0.539); (**B**) One-year mortality ROC: OPS demonstrates strong and significantly higher discrimination than SII (AUC = 0.859 vs. 0.633). (**C**) In-hospital mortality AUC comparison: The correlated ROC curves differed significantly by the DeLong test (*p* < 0.001). (**D**) One-year mortality AUC comparison: DeLong test confirmed significant AUC superiority of OPS over SII (*p* < 0.001). Consistent curve colors across panels represent the same predictors for visual continuity between ROC and AUC comparisons.

In addition, the OPS composite score demonstrated superior discriminatory performance compared with SII alone for both in-hospital mortality (AUC = 0.776 vs. 0.539; ΔAUC = 0.237; DeLong *p* < 0.001; Figure 6A,C) and one-year mortality (AUC = 0.859 vs. 0.633; ΔAUC = 0.226; DeLong *p* < 0.001; Figure 6B,D).

### 3.6. Causes of In-Hospital and One-Year All-Cause Mortality

A total of 28 patients died during hospitalization following TAVI. Of these deaths, 17 were cardiovascular (CV), and 11 were non-cardiovascular (non-CV). The causes of CV death in-hospital included: annular/left ventricular outflow tract rupture (*n* = 2), cardiac tamponade including perforation-related tamponade (*n* = 2), severe acute heart failure (HF) or cardiogenic shock (*n* = 2), major vascular injury or vascular rupture-related bleeding (*n* = 5), progressive renal failure related to CIN (*n* = 4), acute myocardial infarction (*n* = 1), and fatal malignant arrhythmia (*n* = 1), cumulatively totaling 17 CV in-hospital deaths. Non-CV causes of in-hospital death consisted primarily of infection-related complications, including sepsis or septic shock (*n* = 2), pneumonia (*n* = 3), multi-organ failure triggered by non-CV conditions (*n* = 2), acute respiratory failure without direct cardiac etiology (*n* = 3), and ischemic stroke related to non-procedural systemic conditions (*n* = 1), yielding 11 non-CV in-hospital deaths.

During the one-year follow-up period, 53 patients died (all-cause mortality), comprising 27 CV deaths and 26 non-CV deaths. The predominant CV causes of death within one year were progressive HF (*n* = 5), myocardial infarction (*n* = 10), sudden cardiac or arrhythmia-related death (*n* = 8), and ischemic stroke reflecting underlying CV risk burden (*n* = 4), cumulatively equaling 27 CV deaths at one year. The non-CV causes of one-year death were mainly infection-related mortality, including septic or pneumonia-associated death (*n* = 9), chronic respiratory failure of non-cardiac origin (*n* = 7), progressive renal failure unrelated to contrast-induced nephropathy (*n* = 5), frailty-associated functional decline without direct CV etiology (*n* = 3), and other less common non-CV causes (*n* = 2), summing to 26 non-CV deaths at 1 year.

Importantly, the distribution of CV vs. non-CV deaths was relatively balanced, particularly at 1-year follow-up, supporting the representation of both inflammatory–nutritional vulnerability and clinical risk contexts in this cohort.

## 4. Discussion

To the best of our knowledge, this is the first study to demonstrate that the OPS—a composite biomarker comprising CRP, albumin, and TLC—is an independent predictor of both in-hospital and one-year all-cause mortality in patients undergoing TAVI. Our findings indicate that elevated OPS values are significantly associated with worse survival outcomes. Moreover, RCS analysis revealed a nonlinear relationship between OPS and mortality, underscoring the biological plausibility and prognostic strength of this index. In addition, secondary analyses identified vascular complications and established surgical risk models (e.g., EuroSCORE-II) as independent predictors of long-term mortality.

TAVI candidates are typically elderly and burdened with multiple comorbidities, such as frailty, renal dysfunction, and chronic low-grade inflammation [3]. This population is particularly susceptible to adverse outcomes due to diminished physiological reserve, immunosenescence, and malnutrition—factors that frequently coexist and interact deleteriously [3]. These pathophysiologic processes, which are often neglected in traditional risk stratification models, can accelerate cardiovascular decline and impede postoperative recovery. In this regard, systemic inflammation and nutritional status have emerged as central determinants of both short- and long-term outcomes after TAVI [10,11,12]. The inclusion of simple yet biologically relevant markers, such as OPS, which reflect these interrelated domains, may offer pragmatic benefits for preprocedural risk assessment.

The prognostic value of nutritional status in the TAVI population has been previously explored using indices such as the Geriatric Nutritional Risk Index (GNRI) and the Controlling Nutritional Status (CONUT) score. In a prospective study by Lee et al., low GNRI was independently associated with higher one-year mortality, emphasizing the importance of baseline nutritional evaluation [17]. Similar associations were observed in other cohorts, where GNRI consistently predicted post-TAVI mortality [18,19]. A recent meta-analysis also confirmed that low GNRI is associated with increased one-year mortality, while reduced Prognostic Nutritional Index (PNI) values are linked to a higher incidence of major vascular complications [20]. Okuno et al. demonstrated that both CONUT and PNI were significantly associated with increased mortality at one-year follow-up [6]. Likewise, in a retrospective study by Sihag et al. involving 383 patients, GNRI, CONUT, and PNI all independently predicted all-cause mortality [21]. Our findings are consistent with these observations, reinforcing the prognostic roles of hypoalbuminemia and lymphopenia—two OPS components—while also highlighting the added value of incorporating CRP, a marker of systemic inflammation not captured by GNRI, PNI, or CONUT.

Previous studies have also established a link between heightened inflammatory activation and poor prognosis post-TAVI [22,23]. Several inflammation-based indices have been proposed to predict adverse outcomes in this setting. The SII, derived from neutrophil, lymphocyte, and platelet counts, has recently gained attention as a promising marker. Tosu et al. reported that higher preprocedural SII was associated with an increased risk of major adverse cardiovascular events and early mortality, even outperforming CRP in discriminative capacity [24]. In our cohort, however, OPS showed better, statistically significant discrimination than SII for predicting both in-hospital and 1-year all-cause mortality. These results further support the idea that persistent immune dysregulation contributes to the chronic clinical trajectory of post-TAVI patients. Several other studies have similarly identified elevated inflammatory markers as a predictor of adverse outcomes after TAVI [25,26].

OPS offers a comprehensive representation of nutritional and inflammatory status. Elevated CRP and low albumin levels denote a catabolic, pro-inflammatory state that may predispose to procedural complications [27,28], while lymphopenia reflects immune suppression and increased infection risk [29]. Although albumin, lymphocyte count, and CRP each provide prognostic information, they may fail to capture the complex interplay among nutrition, immunity, and inflammation. OPS, by integrating these parameters into a single score, enables a more holistic and synergistic assessment, thereby enhancing prognostic accuracy and supporting clinical decision-making in TAVI patients. CRP is a well-established acute-phase reactant that reflects systemic inflammation, predominantly driven by interleukin-6, tumor necrosis factor-alpha, and interleukin-1β [30]. These cytokines can induce endothelial dysfunction, thrombosis, and myocardial stress [30]. Clinical studies have shown that elevated baseline CRP levels are associated with increased post-TAVI mortality in accordance with these pathophysiological insights [31,32]. Hypoalbuminemia, a marker of malnutrition and chronic inflammation, reflects reduced hepatic protein synthesis and is often linked with systemic cytokine elevation [33]. Low albumin levels may lead to decreased oncotic pressure, fluid overload, impaired wound healing, and suboptimal drug pharmacokinetics—factors that collectively worsen outcomes [33,34]. Although the prognostic implications of isolated serum albumin levels in TAVI are not well established, Bogdan et al. demonstrated a significant association between low baseline albumin and all-cause mortality post-TAVI [35]. Lymphopenia, the third component of OPS, serves as a surrogate of impaired adaptive immunity and is associated with adverse outcomes across various cardiovascular conditions [36]. Surgical stress, activation of the hypothalamic–pituitary–adrenal axis, and sympathetic overactivity can induce lymphocyte apoptosis and redistribution, contributing to immune suppression in the early postprocedural phase [29]. This immunosuppressive milieu predisposes patients to infections and delays tissue healing. Consistent with these mechanisms, our findings showed that higher OPS scores were independently associated with both in-hospital and long-term mortality, even after adjusting for conventional risk models such as EuroSCORE-II. Although the ROC-derived cut-off of ≥2 provides optimal discrimination for our cohort, we acknowledge that OPS thresholds may vary across populations with different baseline characteristics. Importantly, our Kaplan–Meier analyses demonstrated a consistent stepwise increase in both early and one-year mortality across OPS categories (0–3), with OPS 3 showing <50% survival and OPS 0 > 90%, supporting the prognostic value of OPS independent of any specific cut-off. When considered alongside other emerging prognostic indices such as GNRI, PNI, CONUT, and inflammation-based scores like SII, OPS can be conceptualized as a broader biological marker because it simultaneously incorporates components of inflammation, nutrition, and immunity. Although our study did not directly compare OPS with GNRI, PNI, or CONUT within the same cohort, the existing evidence supporting each component of OPS suggests that OPS may represent a biologically coherent and pragmatic option for risk stratification in TAVI candidates.

A particularly noteworthy finding was the nonlinear association between OPS and mortality, revealed through RCS modeling. This gradient relationship supports the hypothesis that malnutrition and inflammation act synergistically rather than independently—an idea also recognized in the literature on frailty and aging [37]. Conventional surgical risk models primarily focus on anatomical and procedural factors [38] and fail to incorporate variables such as frailty, inflammation, or nutritional deficits. The integration of biologically grounded, readily accessible indices, such as OPS, could substantially improve the granularity and accuracy of risk prediction in this vulnerable population. Despite its strong biological relevance, OPS solely reflects systemic inflammatory, nutritional, and immune status and does not capture anatomical or procedural factors—such as aortic root dimensions, vascular access complexity, or valve type selection—that are also known to influence TAVI outcomes [3]. In our multivariable analysis, traditional risk models such as EuroSCORE-II, which incorporate these structural and perioperative elements, remained significant predictors of one-year mortality alongside OPS. Therefore, our findings support a complementary rather than a substitutive role for OPS, suggesting that the most accurate risk stratification may be achieved when OPS is integrated with established clinical and procedural risk assessments. From a practical standpoint, OPS could be readily incorporated into routine preprocedural workflows—particularly alongside EuroSCORE-II and frailty assessments—as its components are already obtained in standard laboratory testing and provide a biological dimension not captured by anatomical or procedural risk models.

In addition to biologically driven risk stratification tools such as OPS, recent technological innovations have also improved TAVI planning and procedural precision. Emerging platforms using artificial intelligence, virtual reality, and mixed-reality imaging allow for enhanced three-dimensional visualization of the aortic root anatomy and more accurate prosthesis sizing. A recent study evaluating holographic mixed reality for TAVI planning demonstrated that fully interactive 3D holographic reconstructions derived from CT imaging can improve the assessment of annulus size, coronary ostia height, valve landing zone geometry, and other anatomic features relevant to device selection and procedural safety [39]. While these technologies support anatomical and technical optimization, OPS provides complementary biological prognostic information, and integrating these modalities may ultimately enable a more comprehensive and personalized approach to TAVI risk assessment.

### Study Limitations

This study has several limitations that warrant consideration. First, its retrospective and single-center design may limit the generalizability of the findings to broader TAVI populations. Although comprehensive data were collected from electronic medical records, the potential for residual confounding and documentation bias cannot be excluded. Second, the sample size, while adequate for statistical power in primary analyses, may still be underpowered to detect subtle differences in certain subgroups or interactions between variables. Third, inflammatory and nutritional biomarkers—including CRP, albumin, and TLC—were measured only at the baseline. The lack of serial assessments precludes the evaluation of dynamic changes in these parameters, which may provide additional prognostic information. Additionally, because OPS does not incorporate anatomical or procedural characteristics, it should be interpreted as a complementary biomarker rather than a comprehensive risk model. Fourth, although we compared OPS with other inflammatory indices, we did not evaluate more recently proposed scores, which might further enhance risk stratification in TAVI patients. In addition, although the sample size was sufficient for the primary analyses, the relatively small cohort may limit the statistical power of subgroup or interaction analyses and, thus, the generalizability of certain secondary findings. Larger, multicenter studies are needed to confirm the external validity of our results. Fifth, cause-specific mortality modeling for OPS could not be performed due to the limited number of events, which restricts statistical power for stable cause-based analyses and poses a risk of overfitting in early mortality subgroups. No standardized phenotypic frailty scale was available in our dataset, and clinical frailty could therefore not be directly compared with OPS in this retrospective cohort. We anticipate that biomarker-derived indices, such as those in our dataset, may be useful for future frailty-focused analyses, but this lies beyond the scope of the present study. Finally, although our findings demonstrate a strong association between OPS and mortality, the observational cohort design precludes causal inference; therefore, the results should be interpreted as prognostic rather than causal, and clinical recommendations should be made with appropriate caution.

## 5. Conclusions

This study demonstrates that the OPS independently predicts both in-hospital and one-year all-cause mortality following TAVI. Our findings underscore the potential of OPS as a biologically meaningful and clinically practical tool for risk stratification in this population. By capturing the interplay between inflammation, malnutrition, and immune dysregulation—factors often overlooked by conventional risk models—OPS offers an integrative approach to mortality prediction. Its ease of use and strong prognostic performance make it a promising adjunct in guiding perioperative management and long-term surveillance. Further prospective validation is warranted to establish its role in routine clinical practice and to explore whether interventions targeting its components could improve patient outcomes.

## Figures and Tables

**Table 1:** Clinical and demographic characteristics of in-hospital survivors vs. non-survivors.

Variables	Survivors (*n* = 216)	Non-Survivors (*n* = 28)	*p*-Value
Age, years	79.2 ± 8.2	85.6 ± 5.4	<0.001
Male gender, *n* (%)	112 (51.9)	10 (35.7)	0.373
Comorbidities			
Hypertension, *n* (%)	176 (81.5)	20 (71.4)	0.373
Diabetes mellitus, *n* (%)	88 (40.7)	6 (21.4)	0.162
Atrial fibrillation, *n* (%)	22 (10.2)	5 (17.8)	0.369
Chronic obstructive pulmonary disease, *n* (%)	24 (11.1)	10 (35.7)	0.012
PCI history, *n* (%)	62 (28.7)	8 (28.6)	0.992
CABG history, *n* (%)	44 (20.6)	9 (32.1)	0.163
Medicines			
ACEi or ARB, *n* (%)	132 (61.1)	16 (57.1)	0.775
Beta-blocker, *n* (%)	108 (50)	14 (50)	1.000
Acetylsalicylic acid, *n* (%)	96 (44.4)	8 (28.6)	0.258
Statin, *n* (%)	72 (33.3)	12 (42.9)	0.480
Anticoagulant, *n* (%)	38 (17.6)	8 (28.6)	0.323
Peri- or post-procedural features			
Valve size, mm	27.3 ± 4.2	27.3 ± 3.2	0.981
Self-expanding valve, *n* (%)	166 (76.9)	20 (71.4)	0.654
Balloon-expandable valve, *n* (%)	50 (23.1)	8 (28.6)	0.654
Vascular complication, *n* (%)	30 (13.6)	14 (50.0)	<0.001
Contrast-induced nephropathy, *n* (%)	22 (10.0)	12 (42.9)	<0.001
Complete atrioventricular block, *n* (%)	30 (15.5)	2 (7.7)	0.291
Echocardiographic findings			
Left ventricular ejection fraction, %	53.0 ± 11.9	51.3 ± 10.5	0.608
Aortic valve mean gradient, mmHg	50.1 ± 11.6	50.8 ± 12.7	0.850
sPAP, mmHg	35 (20–70)	39 (25–60)	0.449
Perioperative risk scores			
STS score	10.2 (4.4–29.7)	12.1 (6.1–26.2)	0.929
EuroSCORE-II	4.6 (1.6–28.5)	4.8 (2.3–15.8)	0.676
Laboratory findings			
White blood cells, ×10^3^/mm^3^	7.4 ± 2.3	8.6 ± 3.3	0.212
Hemoglobin, g/dL	11.5 (6.9–16.0)	9.9 (8.0–13.1)	0.006
Hematocrit, %	36.6 ± 5.9	34.8 ± 6.0	0.273
Platelets, ×10^3^/mm^3^	228.6 ± 83.9	274.2 ± 89.3	0.060
Neutrophils, ×10^3^/mm^3^	4.5 (1.3–7.4)	6.9 (2.2–9.9)	0.051
Lymphocytes, ×10^3^/mm^3^	1.6 (0.4–3.5)	1.0 (0.4–4.8)	0.006
Monocytes, ×10^3^/mm^3^	0.6 (0.01–1.35)	0.8 (0.30–1.04)	0.121
Serum albumin, mg/dL	38.7 (21.0–49.7)	35.3 (28.5–42.3)	0.020
Blood glucose, mg/dL	115 (70–327)	116 (61–231)	0.872
Uric acid, mg/dL	6.6 ± 2.0	7.8 ± 3.1	0.198
Serum creatinine, mg/dL	1.0 (0.4–3.7)	0.9 (0.5–11.3)	0.624
Glomerular filtration rate, mL/min/1.73 m^2^	59.2 ± 21.8	55.9 ± 28.9	0.611
C-reactive protein, mg/L	3.6 (0.1–110.4)	21.8 (1.9–66.0)	<0.001
Serum sodium, mmol/L	139 (127–162)	136 (131–144)	0.165
Serum potassium, mmol/L	4.4 (3.2–6.2)	4.2 (3.1–6.3)	0.388
HbA1c, %	6.0 (4.8–11.7)	5.8 (4.0–13.6)	0.469
Total cholesterol, mg/dL	156 (84–292)	145 (86–206)	0.261
Triglycerides, mg/dL	114 (49–426)	123 (70–198)	0.963
LDL-cholesterol, mg/dL	97 (39–218)	79 (33–147)	0.108
HDL-cholesterol, mg/dL	44 (16–70)	47 (25–65)	0.488
Troponin, ng/mL	22.3 (0.1–67.0)	28.7 (0.1–78.1)	0.130
Osaka Prognostic Score	0.80 ± 0.79	1.92 ± 0.99	<0.001
Systemic immune-inflammation index	2.87 (1.91–4.12)	3.21 (2.03–5.48)	0.462
Follow-up (Days)	6 (2–22)	12 (1–45)	<0.001

Abbreviations: PCI, Percutaneous Coronary Intervention; CABG, Coronary Artery Bypass Graft; ACEi, Angiotensin-Converting Enzyme Inhibitor; ARB, Angiotensin II Receptor Blocker; sPAP, Systolic Pulmonary Artery Pressure; STS, Society of Thoracic Surgeons; HbA1c, Hemoglobin A1c; LDL, Low-Density Lipoprotein; HDL, High-Density Lipoprotein.

**Table 2:** Univariable and multivariable regression analysis for predictors of in-hospital mortality.

Variables	Univariable Regression		Multivariable Regression	
	HR (95% CI)	*p*-Value	HR (95% CI)	*p*-Value
Age	1.129 (1.025–1.242)	0.014	1.821 (1.346–2.292)	0.020
Chronic obstructive pulmonary disease	1.231 (1.101–1.409)	0.019	-	-
Vascular complication	1.158 (1.069–1.364)	<0.001	1.461 (1.208–1.657)	0.025
Contrast-induced nephropathy	1.148 (1.062–1.353)	<0.001	-	-
Hemoglobin	0.660 (0.479–0.911)	0.011	-	-
Osaka Prognostic Score	3.984 (1.978–5.024)	<0.001	2.018 (1.632–2.521)	0.017

Abbreviations: HR, Hazard Ratio; CI, Confidence Interval.

**Table 3:** Baseline characteristics of 1-year survivors vs. non-survivors.

Variables	Survivors (*n* = 191)	Non-Survivors (*n* = 53)	*p*-Value
Age, years	79.0 ± 8.2	83.5 ± 6.8	<0.001
Male gender, *n* (%)	100 (52.3)	20 (37.7)	0.052
Comorbidities			
Hypertension, *n* (%)	153 (80)	40 (75.5)	0.353
Diabetes mellitus, *n* (%)	72 (37.6)	20 (37.7)	0.600
Atrial fibrillation, *n* (%)	18 (9.4)	9 (16.9)	0.351
Chronic obstructive pulmonary disease, *n* (%)	16 (8.3)	18 (34.0)	<0.001
PCI history, *n* (%)	57 (29.8)	15 (28.3)	0.855
CABG history, *n* (%)	41 (21.4)	12 (22.6)	0.883
Medicines			
ACEi or ARB, *n* (%)	121 (63.3)	28 (52.8)	0.159
Beta-blocker, *n* (%)	95 (49.7)	28 (52.8)	0.690
Acetylsalicylic acid, *n* (%)	79 (41.3)	22 (41.5)	0.988
Statin, *n* (%)	68 (35.6)	14 (26.4)	0.212
Anticoagulant, *n* (%)	31 (16.2)	14 (26.4)	0.090
Peri- or post-procedural features			
Valve size, mm	27.2 ± 4.3	27.7 ± 3.1	0.366
Self-expanding valve, *n* (%)	146 (76.4)	41 (77.4)	0.885
Balloon-expandable valve, *n* (%)	45(23.5)	12 (22.6)	0.885
Vascular complication, *n* (%)	22 (11.5)	22 (41.5)	<0.001
Contrast-induced nephropathy, *n* (%)	4 (2.1)	8 (15.1)	<0.001
Complete atrioventricular block, *n* (%)	26 (13.6)	8 (18.6)	0.400
Echocardiographic findings			
Left ventricular ejection fraction, %	53.9 ± 11.1	52.6 ± 9.2	0.428
Aortic valve mean gradient, mmHg	49.2 ± 9.9	50.3 ± 12.0	0.551
sPAP, mmHg	38.2 ± 11.7	40.1 ± 10.9	0.295
Perioperative risk scores			
STS score	9.7 (4.4–29.7)	13.4 (5.9–26.2)	<0.001
EuroSCORE-II	4.3 (1.6–28.5)	6.7 (2.3–21.2)	<0.001
Laboratory findings			
White blood cells, ×10^3^/mm^3^	7.5 ± 2.4	7.6 ± 2.4	0.887
Hemoglobin, g/dL	11.5 (6.9–15.8)	10.5 (7.2–15.3)	0.106
Hematocrit, %	36.7 ± 5.7	35.1 ± 6.6	0.129
Platelets, ×10^3^/mm^3^	230.2 ± 75.9	247.0 ± 93.4	0.321
Neutrophils, ×10^3^/mm^3^	4.5 (1.3–7.4)	6.9 (2.1–9.9)	0.091
Lymphocytes, ×10^3^/mm^3^	1.7 (0.5–4.8)	1.3 (0.4–3.1)	0.001
Monocytes, ×10^3^/mm^3^	0.6 (0.01–1.35)	0.6 (0.20–1.12)	0.158
Serum albumin, mg/dL	38.5 (25.4–49.7)	36.6 (21.0–43.5)	0.004
Blood glucose, mg/dL	119 (70–327)	106 (61–279)	0.102
Uric acid, mg/dL	6.7 ± 2.0	7.3 ± 2.8	0.147
Serum creatinine, mg/dL	1.0 (0.4–3.7)	1.0 (0.4 11.3)	0.128
Glomerular filtration rate, mL/min/1.73 m^2^	60.2 ± 21.8	55.7 ± 24.1	0.115
C-reactive protein, mg/L	6.1 (0.1–101.0)	12.3 (0.4–110.4)	<0.001
Serum sodium, mmol/L	139 (127–162)	138 (131–145)	0.259
Serum potassium, mmol/L	4.4 (3.2–6.2)	4.2 (3.1–6.30)	0.161
HbA1c, %	6.0 (4.8–13.6)	5.8 (5.8–10.1)	0.110
Total cholesterol, mg/dL	166 (93–292)	178 (84–273)	0.739
Triglycerides, mg/dL	119 (49–426)	150 (66–284)	0.199
LDL-cholesterol, mg/dL	98 (48–218)	109 (33–209)	0.853
HDL-cholesterol, mg/dL	44 (23–70)	45 (16–65)	0.228
TSH, μIU/mL	1.6 (0.1–18.3)	1.8 (1.0–8.4)	0.569
Troponin, ng/mL	22.3 (0.1–58.2)	25.3 (0.1–67.1)	0.289
Systemic immune-inflammation index	3.11 (2.02–4.89)	4.96 (3.12–7.94)	0.008
Osaka Prognostic Score	0.86 ± 0.75	1.84 ± 0.84	<0.001

Abbreviations: PCI, Percutaneous Coronary Intervention; CABG, Coronary Artery Bypass Graft; ACEi, Angiotensin-Converting Enzyme Inhibitor; ARB, Angiotensin II Receptor Blocker; sPAP, Systolic Pulmonary Artery Pressure; STS, Society of Thoracic Surgeons; HbA1c, Hemoglobin A1c; LDL, Low-Density Lipoprotein; HDL, High-Density Lipoprotein.

**Table 4:** Univariable and multivariable regression analysis for predictors of 1-year mortality.

Variables	Univariable Regression		Multivariable Regression	
	HR (95% CI)	*p*-Value	HR (95% CI)	*p*-Value
Vascular complication	1.179 (1.089–1.362)	<0.001	1.202 (1.080–1.512)	0.001
Contrast-induced nephropathy	1.174 (1.081–1.373)	<0.001	1.459 (1.173–2.217)	0.117
STS score	1.087 (1.029–1.149)	0.003	1.023 (0.937–1.117)	0.617
EuroSCORE-II	1.115 (1.038–1.199)	0.003	1.104 (1.006–1.211)	0.036
Osaka Prognostic Score	2.954 (1.999–4.366)	<0.001	2.125 (1.300–3.473)	0.003

Abbreviations: HR, Hazard Ratio; CI, Confidence Interval; STS, Society of Thoracic Surgeons.

## Data Availability

Data are available from the corresponding author upon reasonable request.
